# A Peep at Russia, and Home by Sweden

**Published:** 1891-11-14

**Authors:** 


### Short Trips for Short Holidays.

### II.—A Peep at Russia, and Home by Sweden.

(*Concluded.*)

It may be pretty confidently stated that no railways equal the English in speed; but travelling in Russia is more comfortable, not to say more luxurious than in any other country we have yet visited. A Russian railway carriage is really a small room furnished with easy chairs. The back of the chairs may be fixed at any angle, and the seat extended to form a sofa or bed. The distance from St. Petersburg to Moscow is four hundred miles, and the train leaves St. Petersburg at 8 p.m., arriving in Moscow at 10 a.m. The line is one of the straightest in Europe, and there may, perhaps, be some element of truth in the old story about the Emperor Nicholas, the plan and the ruler. The line is very well laid, and this, together with the slow pace at which the trains travel, reduces the vibration to a minimum, so that the journey is performed by night train quite easily.

Dr. Jones, of Earlswood, in one of his interesting papers on Russia tells us that St. Petersburg "may be German, or it may be Belgian, or French, or even English; but it is not Russian." This is literally true. It may be a fine city, although we failed to discover this; but except the language spoken there is nothing really characteristically Russian about it. When, however, the traveller walks out of the station at Moscow, and looks over the Holy City, he feels at once that he is being introduced to a different order of things. Gilt domes, blue cupolas starred with gold, quaint turrets, copper-green roofs, whitened houses meet the eye at every turn. The streets are more vilely paved than those of St. Petersburg, and as myriads of droskis are continually whirling about in all directions, it may be imagined that the noise is terrific. Our carriage stopped at the door of the Hotel Slavianski or the Bazaar Slave as the Russians name it. It is the largest hotel in Moscow—indeed one of the largest in Russia. The hotel porters were on the door-steps, clad in long dark-coloured pelisses and their caps decorated with peacocks' feathers. In the dining-room is a large marble basin in which many fish swim about, so that the intending diner may select his favourite and have it sent to the kitchen.

The place first visited is sure to be the Kremlin; but how can anything so strange and startling and so foreign to our western ideas be described so as to convey even a vague idea of its glories to the mind of the reader? On our first visit the morning sun was pouring out his rays from a cloudless sky, and the glare was almost overwhelming—the Kremlin dazzling in its strange splendour. Imagine a plateau two or three miles in extent rising near the centre of the city to a height considerably above anything near it, the old castellated walls surrounding it; some of the turrets being square; some round—all their roofs bright green.

The Kremlin is really a conglomeration of cathedrals, palaces, towers, and museums. No order is apparent in their arrangement. The royal palace is prominent by reason of its enormous size, but it looks plain and commonplace when contrasted with the buildings at its side. One of the cathedrals has a huge central dome and a number of smaller ones grouped around as if to support the larger, and all are gilt and glancing in this brilliant sunshine—intercepting the rays, but only to throw them off again in lines of dazzling light. In another the central dome only is golden while the others are blue. Yet another has its roof bristling with cupolas and minarets, every point gleaming and sparkling as if set with diamonds. A fourth has its domes painted of so curious a dark blue that we are lost in wonder as to how that colour could have been fixed. There are pinnacles and minarets whose numbers one must not try to reckon. Nearly all the domes and cupolas are surmounted by crosses, and oftentimes the cross rests on a crescent, either showing the Eastern origin of much that we see around, or signifying that the crescent has waned there for ever. Rising high above the others are the tower of Ivan Veliki and the spire over the Redeemer's Gate. The tower of Ivan Veliki is "telescopic" in construction, and the parapet of each story is wreathed with bells. It was from this tower that Madame de Stael looked on the magnificent glittering panorama at her feet and said, "Behold the Rome of the Tartars." It was here, too, that Napoleon saw the city burning around him, and felt that his hopes of conquering Russia were doomed At the foot of the tower is the great Bell of Moscow—the Czar Kolokol. The Redeemer's Gate is the chief entrance to the Kremlin. Over the archway is a painting of the Redeemer of Smolensk with its sacred lights burning perpetually. Here thousands of people come every day, cross themselves, and go; and everyone passing under the archway has to take off his hat. Now we are within the Kremlin walls—hats are on again, and a short walk takes us to the Cathedral of the Rest of the Virgin. It is in this church that the Czars are crowned. Outside it looks plain except for the cupolas; but inside it is a blaze of gilding and colours. The frescoes taken individually do not strike one as anything remarkable, but the effect of the whole is striking and indescribable. The Iconostas blazes with gold and jewels. One picture of the Virgin is hung round with brilliants, emeralds and rubies, said to be worth £150,000. A service in this church must indeed be an imposing spectacle. Here is a description of what takes place at midnight on Easter eve. "The great bell sounds, followed by every other bell in Moscow; the whole city blazes into light; the tower of Ivan Veliki is illuminated from its foundation to the cross on its summit. The square below is filled with a motley throng, and around the churches are piles of Easter cakes each with a taper stuck in, waiting for a blessing. The interior of the church is thronged by a vast multitude bearing wax tapers. The Metropolitan and his clergy in robes blazing with gold and precious stones have made the external circuit of the church three times, and then through the great doors have advanced towards the throne between myriads of lights. No words can describe the colour, the blaze, the roar of the universal chaunt." After that, when meetings take place, one says, "Christos vcscres," and the other answers, "Vo istine voscrés."

In another cathedral the Czars are married, and in the cathedral of the Archangel Michael they were buried until the time of Peter the Great. One of the most touching tombs is that of the young Dimitri. It is in a small shrine approached by two or three steps. The lid of the outer coffin is thrown open so that the inner may be kissed by those who visit it, and the poor little murdered boy's clothes hang at one end.

Outside the Kremlin and nearly opposite the Redeemer's Gate stands what is perhaps the most extraordinary looking Christian church in the world—that of St. Basil the Beatified. It was built by Ivan the Terrible in 1554. The cupolas are of different shapes, and the stones composing them are of various forms and variously sculptured. Some are smooth, others ribbed. Fluted ones are seen in one place, and parallels and spirals in another. The colouring is also varied, and bright to a degree not elsewhere to be seen.

The new Cathedral of our Saviour with its white marble fronts, its beautiful proportions, its lovely internal decorations, shows that even the nineteenth century can produce a cathedral.

A week or more might be profitably spent in and around Moscow; but not more than three days can be spared from this "short holiday," so we make our return journey to St. Petersburg on our way to Finland and Sweden. The irritating and unnecessary passport formalities have been gone through, and the ticket obtained for a passage to Stockholm. The steamers leave about five in the afternoon. They are Swedish, extremely clean, and comfortable in every way. Helsingfors is reached in the morning. The approach is very fine. The little islands and the mainland form a sort of amphitheatre or natural harbour, which has been improved by well constructed quays for large vessels, and tiny docks for small ones. The town is clean and pretty, abounding n open places thickly planted with trees. The inhabitants are almost all Lutheran in religion, while the garrison and the fleet are of the Russian Church. A drive of one hour's duration will show the visitor something of the suburbs, and give him a glance at the pine woods. On his return he can rest at the Brunnsparken. From Helsingfors to Hango takes about seven hours, and a charming little voyage it is. The steamer threads its way along a narrow arm of the gulf. Sometimes this expands into a lake-like sheet of water, studded with islets, many of them covered with trees, and others consisting only of huge grey rocks. The next place touched is Abo, after which the Alland Isles are passed; then the Gulf of Bothnia is crossed, the course now being amid the three thousand islets which guard the entrance to Stockholm. The entire voyage, including stoppages, takes nearly three days. Stockholm is indisputably one of the finest cities in Europe, and may fairly claim its second name, "Venice of the North." A few days will suffice for seeing it and for a trip to Lake Malaren. The traveller may cross Sweden by train or by canal, or by a combination of these, to Gotenburg. There he will get steamers to Hull twice a week; one leaves at mid-day on Fridays, and consequently lands its passengers early on Sunday morning in Hull. Why it should be timed to reach England on a Sunday, when the train service is suffering from universal dislocation, is a mystery. The Gotenburg boats are much better than the St. Petersburg ones. A curious custom prevails in them of charging food separate from the passage, and it has to be paid for whether consumed or not, which is rather hard on those who are sick all the voyage. For ourselves, not being lunch eaters, we asked for a cup of tea either before the luncheon hour, or at lunch, or shortly afterwards, but we were told it could not be supplied until half-past four, which, we say again, is rather hard.

Why one of the railway companies doesn't run a line of really good steamers to Russia, Sweden, and Norway during the season is yet another mystery. We commend this idea to the Hull and Barnsley directors.

